# Management of a Traumatic Bone Cyst of the Mandible: A Case Report

**DOI:** 10.7759/cureus.109614

**Published:** 2026-05-25

**Authors:** Saurabh Dudhe, Pragati Nivruttirao Jadhav, Jay Goyal, Shruti S Joshi, Shyamali Lahon

**Affiliations:** 1 Department of Oral and Maxillofacial Surgery, Jawahar Medical Foundation's Annasaheb Chudaman Patil Memorial Dental College, Dhule, IND; 2 Department of Oral and Maxillofacial Surgery, Terna Dental College and Hospital, Navi Mumbai, IND; 3 Department of Oral and Maxillofacial Surgery, DJ College of Dental Sciences and Research, Ghaziabad, IND

**Keywords:** anterior mandible, curettage, cystic lesion, dystrophic calcification, root resorption, surgical curettage, traumatic bone cyst

## Abstract

Cystic lesions of the anterior mandible in young adults present a diagnostic challenge owing to the overlapping clinical and radiographic features of odontogenic keratocysts, unicystic ameloblastomas, central giant cell lesions, and traumatic bone cysts. A 25-year-old man presented with localized pain and swelling of the anterior mandible. The left mandibular first premolar was nonvital, and panoramic radiography revealed a 2 × 3 cm well-defined radiolucent lesion with sclerotic, scalloped margins and associated external root resorption. Fine-needle aspiration cytology did not yield any fluid (dry tap). Surgical exploration revealed an empty bone cavity with minimal straw-colored fluid and no discernible epithelial linings. Histopathological examination revealed fibrous connective tissue with chronic inflammation, dystrophic calcification, and focal multinucleated giant cells, but no epithelial lining. The final diagnosis was a traumatic bone cyst of the mandible. Surgical curettage with bleeding stimulation was performed. This case underscores the need for surgical exploration and histopathology to make a definitive diagnosis, as radiographic and cytological findings alone are insufficient to distinguish traumatic bone cysts from other cystic lesions.

## Introduction

Cystic lesions in the jaw represent a diagnostically challenging subset of oral and maxillofacial pathology. Owing to its rich odontogenic epithelial remnants and complex developmental history, the mandible is particularly predisposed to a diverse spectrum of cystic conditions, ranging from common odontogenic cysts to rare non-odontogenic and pseudocystic entities [1,2]. Traumatic bone cysts are uncommon non-epithelial intraosseous lesions, accounting for approximately 1% of jaw cysts, with a higher predilection for the mandible and younger individuals [1]. When such lesions are present in the anterior mandible of a young individual, the differential diagnosis broadens considerably, demanding meticulous clinical, radiographic, and histopathological correlations to arrive at a definitive diagnosis [3].

Jaw cysts are broadly classified into two categories: odontogenic and non-odontogenic. Among odontogenic cysts, dentigerous cysts, odontogenic keratocysts (OKCs) (now reclassified as keratocystic odontogenic tumors in certain contexts), and radicular cysts are the most frequently encountered lesions [4]. Non-odontogenic cysts, including traumatic bone cysts and aneurysmal bone cysts, although less common, must also be considered in the anterior mandibular region [5]. Compounding the diagnostic challenge is the reality that many of these entities share overlapping clinical and radiographic features, rendering a diagnosis based solely on imaging unreliable and potentially misleading.

The anterior mandible presents a unique anatomical milieu in which developmental, inflammatory, and neoplastic processes may converge [6]. Bony swelling in this region in young adults may mimic a variety of benign lesions and, in rare instances, may harbor more aggressive pathologies, such as ameloblastoma or central giant cell granuloma [7]. The clinical presentation of a slow-growing, asymptomatic bony expansion is common across multiple diagnoses, highlighting the critical need for a thorough diagnostic evaluation. This report presents the case of a 25-year-old man who presented with bony swelling of the anterior mandible and highlights the diagnostic complexity of such cases. Through this case, we aim to emphasize the importance of a systematic multimodal diagnostic approach in distinguishing between entities that may closely simulate one another and guide appropriate and timely management.

## Case presentation

A 25‑year‑old man presented with localized pain and tenderness in the anterior mandible. He reported no relevant medical history or significant episodes of trauma to the chin or mandible.

Extraoral clinical photograph revealed mild swelling over the lower border of the anterior mandible, localized to the left side. Intraoral examination revealed no mucosal abnormalities such as obliteration of the left mandibular buccal vestibule; however, expansion of the buccal cortical plate was noted. Although the teeth in the lesional area were free of caries, the lower left first premolar was nonvital on thermal test and tender on percussion. Given the intrabony nature of the lesion, routine hematological and biochemical investigations were normal. No specific laboratory abnormalities were observed (Table 1).

**Table 1:** Laboratory investigations

Test	Patient value	Reference range
Hemoglobin	12.4 g/dL	Male 13–18 g/dL; female: 12–16 g/dL
Fasting Plasma Glucose	76 mg/dL	< 100 mg/dL
Serum Creatinine	0.85 mg/dL	Male: 0.7–1.2 mg/dL; female: 0.5–1.0 mg/dL
Serum Calcium	9.6 mg/dL	8.5–10.5 mg/dL
Serum Alkaline Phosphate	76 IU/L	44–147 IU/L

A panoramic radiograph revealed a well-defined radiolucent lesion measuring 2 × 3 cm with a sclerotic (well-corticated) border and scalloped margins located in the anterior mandible and associated with the left lower first premolar. Importantly, the radiograph also showed external root resorption of the first premolar due to the expansion of the cyst. The pathological migration of the tooth created a space between the left mandibular first and second premolar (Figure 1).

**Figure 1:**
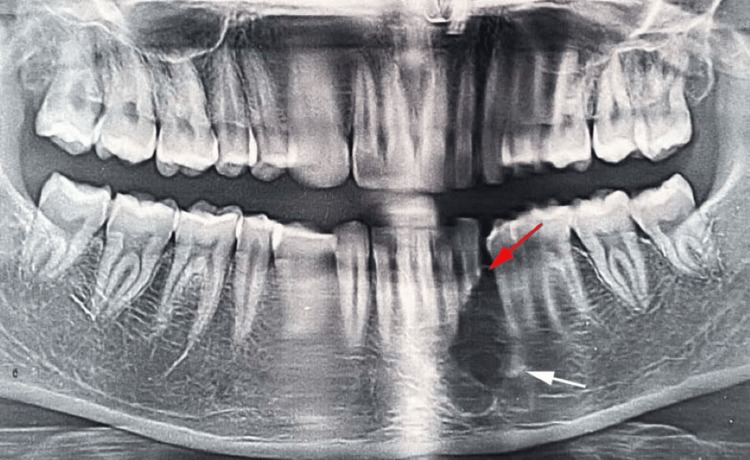
Panoramic radiograph of the anterior mandible demonstrating a well-defined radiolucent lesion with a corticated (sclerotic) border and scalloped margins. The white arrow indicates the radiolucent cystic area, and the red arrow indicates root resorption of the left mandibular first premolar.

Fine-needle aspiration cytology (FNAC) was performed on the lesions as a minimally invasive diagnostic method. No fluid was obtained during the aspiration. Based on the radiographic findings of a well-defined radiolucent lesion with a sclerotic border, root resorption of the lower left first premolar and vital adjacent teeth, and a dry tap on FNAC, the differential diagnoses included OKC, unicystic ameloblastoma, central giant cell lesion, and traumatic bone cyst. The absence of aspirated fluid suggested a solid or empty lesion, with OKC and traumatic bone cysts being the most likely considerations.

Surgical exploration was followed by thorough curettage of the bony walls. This procedure has both diagnostic and therapeutic applications. In the present case, under local anesthesia, a full-thickness mucoperiosteal flap was raised to expose the buccal cortical plate, which was found to be expanded but intact. A bony window was created over the lesion, revealing an empty bone cavity with no discernible epithelial lining and minimal straw-colored fluid (Figure 2A). The cavity was aggressively curetted with a Lucas surgical curette to remove all fibrous tissue, thereby stimulating fresh bleeding from the defect. Adjacent resorbed teeth were also extracted. The flap was then sutured back into place (Figure 2B). 

**Figure 2:**
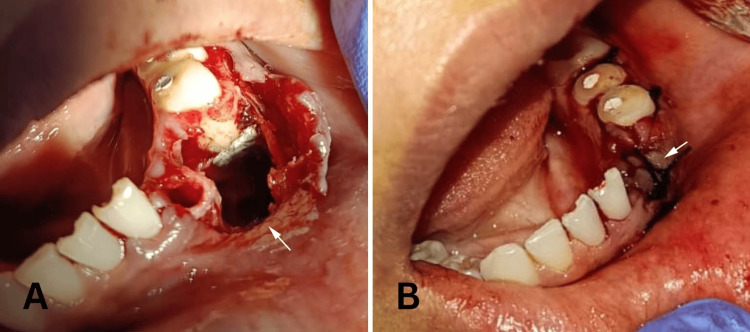
Intraoperative photographs showing (A) creation of a bony window in the buccal cortical plate revealing an empty bone cavity (white arrow), and (B) closure following curettage and extraction of the involved tooth, with sutured mucoperiosteal flap (white arrow).

Given the inconclusive FNAC results and atypical radiographic features (sclerotic border and root resorption), surgical exploration was performed, and a tissue sample was obtained for biopsy. The curetted material consisted of fragments of soft fibrous tissue with no definite cyst wall. Histological sections revealed loose fibrous connective tissue with scattered foci of chronic inflammatory cells. Small foci of dystrophic calcification and islands of new bone formation were also observed within the fibrous walls (Figure 3A). Multinucleated giant cells were present in the deeper regions (Figure 3B). No epithelial lining was identified in any of the sections.

**Figure 3:**
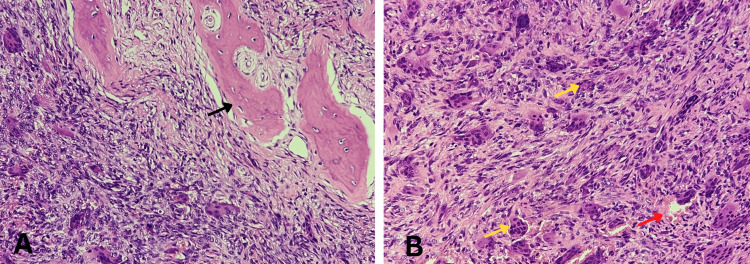
Histopathological images (hematoxylin and eosin stain, x40 magnification) showing (A) loose fibrous connective tissue with focal dystrophic calcification and areas of new bone formation (black arrow), and (B) presence of multinucleated giant cells (yellow arrow) and red blood cells (red arrow) within the deeper connective tissue. No epithelial lining is identified.

After surgery, the blood clot that forms in the involved tooth is organized, as specialized cells (mesenchymal cells like osteoblasts) migrate into the area. Osteoblasts (bone-building cells) deposit a soft matrix called osteoid. This matrix gradually mineralizes and hardens with calcium and phosphate, transforming into woven bone. The case was followed up for six months with no recurrence. 

## Discussion

The present case posed a considerable diagnostic challenge owing to the convergence of overlapping clinical, radiographic, and cytological findings that did not conform exclusively to a single pathological entity. The principal diagnostic challenge in the present case was differentiating the lesion from other odontogenic cysts and benign radiolucent jaw lesions based solely on clinical and radiographic findings. The lesion appeared as a well-circumscribed radiolucency with minimal symptoms, which could resemble entities such as odontogenic keratocyst, unicystic ameloblastoma, residual cyst, or central giant cell lesion. Therefore, a definitive diagnosis required intraoperative findings and histopathological correlation. The differential diagnosis in this case, encompassing an OKC, unicystic ameloblastoma, central giant cell lesion, and traumatic bone cyst, reflects the well-documented reality that anterior mandibular lesions in young adults share considerable radiographic mimicry, making histopathological confirmation essential.

The vitality status of teeth associated with jaw lesions has significant diagnostic weight. In the present case, the left lower first premolar was nonvital and tender on percussion, whereas the remaining teeth in the lesional area retained their vitality. This finding is of particular diagnostic relevance, as odontogenic cysts of inflammatory origin, such as radicular cysts, are invariably associated with nonvital teeth and derive their epithelial lining from the proliferation of the rest of Malassez in response to periapical inflammation [8]. However, developmental cysts, such as OKC and dentigerous cysts, typically arise in association with vital teeth, and their presence adjacent to a nonvital tooth is considered atypical [9]. The lack of vitality of the first premolar in this case, in the absence of caries or obvious trauma, may represent a coincidental finding or reflect pressure-induced vascular compromise secondary to the expanding lesion.

The radiographic appearance of a well-corticated, scalloped radiolucency is classically associated with OKC, a lesion known for its aggressive clinical behavior, high recurrence rate, and propensity for root resorption. OKC frequently presents as unilocular or multilocular radiolucency with corticated margins in the mandible of young patients [2]. Root resorption, observed in the present case involving the first premolar, is a notable radiographic finding that requires careful consideration. While root resorption is most characteristically and extensively associated with ameloblastoma, it has also been reported in OKC, central giant cell lesion, and traumatic bone cyst, albeit less frequently [1,4]. Although uncommon, root resorption in traumatic bone cyst can occur when the lesion exerts sustained pressure against the root apex over a prolonged period, particularly in younger patients in whom the root structure is still maturing [10]. Therefore, this finding cannot be used exclusively to favor or exclude any diagnosis in the present case. However, the definitive diagnosis of OKC rests on the histopathological identification of a thin, parakeratinized, corrugated epithelial lining, a feature conspicuously absent in the present case [11]. Unicystic ameloblastoma, another important differential diagnosis, typically presents as a well-defined unilocular radiolucency and is known to cause significant root resorption, as highlighted by Ramesh et al. [12] in their study. The absence of epithelial lining on histology effectively excluded both OKC and unicystic ameloblastoma from the final diagnosis.

Central giant cell lesions warrant consideration, given the patient’s age and the location of the mandible. Eid et al. described central giant cell lesion as a lesion with bluish color surface and capable of presenting with cortical expansion and root resorption [6], which were features present in this case. However, central giant cell lesion is histologically characterized by a cellular fibroblastic stroma rich in multinucleated giant cells distributed throughout the lesion rather than focally. In the present case, multinucleated giant cells were identified only in the deeper portions of the fibrous wall, a distribution more consistent with a reparative response than a primary giant cell lesion, thereby reducing the likelihood of central giant cell lesion as the definitive diagnosis [13].

The final clinicopathological diagnosis was a traumatic bone cyst, a pseudocyst of debated etiopathogenesis. The absence of epithelial lining is a pathognomonic histological feature of traumatic bone cyst that fundamentally distinguishes it from true cysts of the jaw. Unlike odontogenic cysts, which possess an epithelial lining derived from odontogenic remnants, traumatic bone cyst represents a pseudocystic cavity lined only by a thin layer of loose fibrous connective tissue or, in many instances, by no discernible soft tissue [7]. This critical absence of epithelium, consistently observed across all submitted histological sections in the present case, has been established as a definitive diagnostic criterion for traumatic bone cyst in multiple studies [7,14].

The hallmark intraoperative finding of an empty bony cavity with minimal straw-colored fluid further reinforces this diagnosis. The absence of aspirable fluid on FNAC, initially a confounding finding, is entirely compatible with traumatic bone cyst. The fluid content of traumatic bone cyst is highly variable, and many lesions contain little to no fluid at the time of aspiration, particularly in the earlier or more quiescent stages of the lesion's evolution. Goyal et al. noted that the aspiration yield in traumatic bone cyst is unpredictable and should not be used as the sole criterion to exclude the diagnosis, as a dry tap may simply reflect an empty or near-empty cavity rather than a solid neoplastic process [15]. The presence of scattered chronic inflammatory cells, dystrophic calcification, and nascent bone formation in the fibrous wall, as observed in this case, has also been documented by Tariq et al. [16] and reflects the reparative biological activity occurring within the cavity. Surgical exploration with aggressive curettage to stimulate bleeding and subsequent clot organization remains the most widely accepted and effective therapeutic modality for traumatic bone cyst, with reported resolution rates exceeding 90% in long-term follow-up studies [7].

An additional histopathological finding in the present case was the presence of multinucleated giant cells in the deeper portion of the lesion. Although such cells may occasionally raise concern regarding the possibility of recurrence or association with other giant cell lesions, focal multinucleated giant cells have also been reported as reactive findings in traumatic bone cysts [14]. In the present case, the overall clinicoradiographic and histopathological correlation supported the diagnosis of traumatic bone cyst rather than an aggressive giant cell lesion. Nevertheless, considering this finding, long-term periodic follow-up was advised to monitor bone healing and detect any possible recurrence.

## Conclusions

Traumatic bone cysts may occasionally present with atypical features such as root resorption, non-vital tooth association, and localized cortical expansion, thereby mimicking more aggressive odontogenic lesions. This case highlights the importance of a systematic diagnostic approach integrating clinical examination, vitality testing, radiographic evaluation, aspiration findings, surgical exploration, and histopathological correlation for accurate diagnosis. Surgical exploration with curettage remains both diagnostic and therapeutic in traumatic bone cysts, while periodic follow-up is essential to monitor healing and detect possible recurrence.
